# How to Report Systematic Review Studies; A Critical Review of Some Methodological Issues and Recommendations to Consider When Conducting These Studies

**DOI:** 10.29252/beat-070416

**Published:** 2019-10

**Authors:** Mehrdad Amir-Behghadami, Ali Janati

**Affiliations:** 1 *Iranian Center of Excellence in Health Management (IceHM), Department of Health Service Management, School of Management and Medical Informatics, Tabriz University of Medical Sciences, Tabriz, Iran*; 2 *Student Research Committee (SRC), Tabriz University of Medical Sciences, Tabriz, Iran*

**Keywords:** Systematic Review, Methodology, Critical Review

***Dear editor,***

Recently a study titled, “A Productive Proposed Search Syntax for Health Disaster Preparedness Research”, was worked by Rastegarfar *et al*. in the Bulletin of Emergency And Trauma [1]. First of all, we would like to thank the editors that help to appear review study. In addition, we would like to extend our appreciation to the authors of this article. Review studies are considered as studies with the highest level of evidence that play an important role in evidence-based decision making [2]. The results of the mentioned study are an interesting; however, we believe that there are some questions regarding the study, which, we would like to present. These questions, if replied, will only apply to improve the quality of the current study and similar studies in the near future.

Based on the explanations given in method, this study seems to be a systematic review if so; it should be included in the study title of the word "systematic review". We would like to raise a question about the rationale behind not searching for other national and international databases. In a systematic review, all studies conducted in one scope must be identified so searching one or two databases is inadequate and can decrease sensitivity to as low as 66% [3]. However, the authors have searched only an electronic database, PubMed. Inclusion and exclusion need to be much more clarified in detail and it isn’t clear whether papers were expected to meet all of these criteria or any of these criteria. Although excluding non-English articles reduces the power of the article it should be mentioned in exclusion criteria. We also would like to ask what kind of studies have been included or excluded in this review. The PRISMA flow has been not plotted for the retrieved studies. In accordance with the PRISMA guidelines, the PRISMA flow could be plotted and presented in the results section. Since assessing the quality of the included studies is necessary, it is better to mention the characteristics of the articles according to the quality evaluation score in the results section. We would like to ask whether not appraising the quality of the included studies was an oversight or an intentional decision, and if so, what argument is behind it. Poor-quality studies will affect the quality of the results and will distort the results of the studies. The Joanna Briggs Institute (JBI) is an international Institute that aims to promote evidence-based health services by providing access to health-related resources [4]. The JBI Critical Appraisal Checklist has been designed and approved by the JBI Scientific Committee. It is recommended that these checklists be used in evaluating the quality of studies, as specific checklists have been designed for a wide range of studies. [5]

Hence, in order to be more clarity, it is recommended that researchers conducted their studies in accordance with PRISMA statement so, its use decrease the risk of flawed reporting and increase its quality. [5-7] Despite the good findings, it seems that the method of this study has not been properly reported and has been not clearly stated for better understanding of readers. Answering these questions may inform future research to prevent such mistakes being made clear.
